# Randomized Controlled Trial Considering Varied Exercises for Reducing Proactive Memory Interference

**DOI:** 10.3390/jcm7060147

**Published:** 2018-06-11

**Authors:** Emily Frith, Eveleen Sng, Paul D. Loprinzi

**Affiliations:** 1 Physical Activity Epidemiology Laboratory, Exercise Psychology Laboratory, Department of Health, Exercise Science and Recreation Management, The University of Mississippi, MS 38677, USA; efrith@go.olemiss.edu; 2 Jackson Heart Study Vanguard Center at Oxford, Physical Activity Epidemiology Laboratory, Exercise Psychology Laboratory, Department of Health, Exercise Science and Recreation Management, The University of Mississippi, MS 38677, USA; ssng@go.olemiss.edu

**Keywords:** learning, memory consolidation, memory retrieval, physical activity, verbal recall

## Abstract

We evaluated the effects of exercise on proactive memory interference. Study 1 (*n* = 88) employed a 15-min treadmill walking protocol, while Study 2 (*n* = 88) included a 15-min bout of progressive maximal exertion treadmill exercise. Each study included four distinct groups, in which groups of 22 participants each were randomly assigned to: (a) exercise before memory encoding, (b) a control group with no exercise, (c) exercise during memory encoding, and (d) exercise after memory encoding (i.e., during memory consolidation). We used the Rey Auditory Verbal Learning Test (RAVLT) to assess proactive memory interference. In both studies, the group that exercised prior to memory encoding recalled the most words from list B (distractor list) of the RAVLT, though group differences were not statistically significant for Study 1 (walking exercise) (*p* = 0.521) or Study 2 (high-intensity exercise) (*p* = 0.068). In this sample of young adults, high intensity exercise prior to memory encoding showed a non-significant tendency to attenuate impairments in recall attributable to proactive memory interference. Thus, future work with larger samples is needed to clarify potential beneficial effects of exercise for reducing proactive memory interference.

## 1. Introduction

### 1.1. What Is Proactive Interference?

Proactive interference (PI) may be defined as a fundamental inefficiency to generate or converge on accurate responses following the presentation of novel learning stimuli. For example, if exposed to and asked to learn (recall) two successive word lists (List A and B), the performance on the second list (List B) may be hindered by the proactive interference effect of having to learn the first list (List A). Retroactive interference, on the other hand, would be defined by the deleterious effects on learning List A caused by the need to learn List B.

The risk of PI is exacerbated when learning and target stimuli share similar attributes, and when the volume of information increases preceding the salient target [1]. Recall accuracy may also be directly influenced by the semantic similarity of the stimuli. When learned materials resemble new material (i.e., word lists), the rate of memory-trace degradation and convergence error is magnified [1]. Research suggests retention is inversely proportional to the number of precursory exposure trials [2]. Thus, an influx of ambiguously encoded content renders effective discernment of target response cues increasingly difficult.

### 1.2. Theoretical Overview

The Multicomponent Theory [2] of retrieval posits that memory traces are, in essence, a systematically organized vector of items. Simplistically, the possibility for successful recall is dependent upon adequate encoding. However, semantic trace equivalency produces a litany of convergence issues, wherein mental representations of verbal cues may cause incorrect answers to seem equally and semantically correct [1]. Additionally, as the length of storage time between encoding and recall increases, this issue of ambiguity becomes especially detrimental to memory processing. Regarding temporal spacing effects, when a recall test occurs immediately after encoding, the retention interval does not appear to be significant to induce deleterious PI on memory retrieval [3]. The Multicomponent Theory accounts for the gradual decay of specificity, as vectors of equivalent content become mentally indistinguishable [1]. Importantly, this loss is more gradual when the composition of accurate responses is entirely distinct from previously encoded information (i.e., learning a novel target word list after exposure to a prior word list) [1].

Trace equivalence is merely one aspect underlying the complexities influencing PI effects on short-term memory. Another potential moderator of recall accuracy is trace-selection failure, wherein errors manifest as an inability to isolate correct responses from remembered items, rather than deciphering the meaning of the composite trace [1]. For clarity regarding the equivalency component of trace-decay, a participant might derive a response from memory trace residuals that are subject to PI as a function of intruding amalgamated items [2]. Alternatively, trace-selection denotes a PI-induced decrement in encoding quality, as memory maintenance across the experimental condition is compromised [4]. Correct responses are not only manufactured by collapsing similar information into short-term memory storage, but may be formed from internal editing [1]. Erroneous lexical cues may be implicitly screened prior to recall, suggesting that both short-term, event-related cues, as well as vocabulary in long-term storage are prone to trace-selection interference. For example, according to Bennett, “…the word “TEST” would be reported when the to-be-remembered-item (TBRI) had been the word “BEST”; “BEST” would never be replaced with “BESP” or “BESB”, even though these responses also represent simple one-phoneme transformations of the original stimulus term [1].”

### 1.3. Biological Overview

Following a brief discussion of classic theoretical positions, it is important to provide a snapshot of the underlying cognitive mechanisms delineating PI. Recent work indicates the medial prefrontal cortex (mPFC) plays a critical role in hippocampal encoding of salient task criterion, serving as a combatant safeguard against competing stimuli [5]. The mPFC differentiates memory cues at the point of encoding, which assists correct retrieval [5]. Multiple authors support the position that the prefrontal cortex is activated during semantic processing of new content susceptible to distortion from previously encoded information [5,6,7,8,9,10]. In fact, the prefrontal cortex may also be quite sensitive to novel stimuli [8,11]. Moreover, prefrontal lesions are shown to amplify PI [7,9]. These damaging neuronal effects have been corroborated by slower convergence speed and increased rate of subject response error [10]. Additional evidence proposes that a complex interplay of cognitive processes operate in harmony to evaluate and monitor both previously learned, and newly experienced stimuli, facilitating successful processing across time [12].

In addition to the mPFC, the bilateral fronto-polar cortex (FPC) aids cognitive control concerning mnemonic cues for list membership [13,14,15,16]. Both familiar and recently encoded information is evaluated according to event-dependent cognitive criteria [12,13,15]. Cognitive conflict buffers against PI do not seem to be confined to the mPFC and bilateral FPC. Neuroimaging data provides plausibility for regulatory mechanisms to extend to the left FPC and left mid-ventrolateral PFC as well [13,14,15,16].

### 1.4. Exercise and PI

It is well established that physical activity increases the release of neurotransmitters implicated in the formation of new memories [17,18]. That is, the rise in catecholamine levels during exercise may assist optimal memory encoding and consolidation [19]. Exercise-induced arousal is thought to facilitate attentional allocation necessary for encoding processes, which could be protective against PI during initial learning. Exogenous epinephrine, an excitatory neurotransmitter, has been shown to enhance memory capacity before and after learning [20]. McNerney detected similar biologically-induced results before and after acute exercise alone on visual, sentence recognition tasks and declarative, text-based tasks [20]. Favorable memory effects were maintained one week later, suggesting exercise is preferable to no exercise regarding both encoding and consolidation learning processes [20]. Beyond neurotransmitter release, exercise may increase cortical brain volume [21], support prefrontal and mPFC growth effects modulating synaptic, dendritic and astrocytic plasticity [22], and facilitate the expression of specific neurotropic growth factors, such as BDNF. BDNF is expected to alter hippocampal structures, promoting efficient neurotransmission and synaptic plasticity [23]. Further, exercise-associated affect influences cognition and memory consolidation [24]. Physical activity also impacts executive functioning [24,25,26], and inhibitory control, partially driven by activation of the anterior cingulate cortex [27].

Although emerging work has identified the potential for acute exercise to influence brain activation in regions specifically involved in PI control, there is a paucity of empirical evidence demonstrating a specific association between exercise and PI. In fact, we are unaware of a study specifically addressing this issue. Therefore, we examined the possibility that acute exercise would reduce PI. We manipulated the timing of exercise in relation to list learning [28] (i.e., exercise before, during or after learning) to study what role these varied temporal effects might play in PI related learning and retention. We also investigated the distinct effects of aerobic exercise intensity on encoding processes. As discussed previously, exercise-induced arousal may influence memory retention [29]. Moderate intensity exercise is proposed to boost memory, as intermediate physical efforts are unlikely to impose physiological stressors sufficient to remove PFC allocation resources [30]. To our knowledge, no study has evaluated the potential intensity-specific and temporal effects of exercise in reducing proactive interference effects on memory. We specifically hypothesize that exercise prior to memory encoding (compared to exercise during encoding and the control group) will have the greatest effect in memory performance after a PI stimuli. Further, with regard to exercise intensity, we hypothesize that the moderate intensity exercise protocol employed in Study 1 (Walking and Learning) will have a greater memory performance effect in the presence of proactive memory interference, relative to the high-intensity exercise protocol followed in Study 2 (Jogging and Learning).

## 2. Method

### 2.1. Participants

A total of one hundred seventy-six healthy young adults were recruited via convenience sampling for both Study 1 (*n* = 88) and Study 2 (*n* = 88). Within each of these studies, there were four exercise groups (*n* = 22 in each group) into which participants were randomized (using a random number generator): (a) exercise before learning, (b) no exercise control group, (c) exercise during learning, and (d) exercise after learning. We chose a participant group size of 22 participants, based on our previous experimental work on this topic [31]. Participants were deemed eligible for the study if they were not pregnant, answered “no” to all questions on the PAR-Q (Physical Activity Readiness Questionnaire), consumed no caffeine within three hours of their visit to the lab, consumed no alcohol nor engaged in any exercise within six hours of their visit to the lab, were not under the influence of any drugs or medications that could alter their mental state, were non-smokers, and had no concussion within the last month. All participants gave written informed consent prior to engaging in any study activity.

### 2.2. Study Design

This study was approved by the Institutional Review Board at the author’s institution. The present data was extracted from two experimental studies (detailed below) that evaluated the effects of varied temporal placement of acute exercise on learning and memory functioning, using the Rey Auditory Verbal Learning Task (RAVLT) to measure PI effects. The RAVLT requires the participant to listen to List A (15 nouns) in five consecutive trials, with free recall occurring immediately after each trial. Then, immediately following the fifth trial, the examinee listens to List B (15 different nouns) with free recall occurring promptly after List B is heard. In the present study, we used the performance on List B of the RAVLT as the outcome of interest, with five trials of List A serving to induce PI to List B recall.

### 2.3. Study 1: Walking

For Study 1, 88 participants had a mean age of 23.3 (SD = 3.7) years. As shown in Table 1, most participants identified as Caucasian (65.9%), while others identified as African American (20.5%), Asian (12.5%), and other (1.1%). This sample was evenly distributed with regard to gender (52.3% males and 47.7% as females). The participants’ mean body mass index (BMI) was 25.3 kg/m^2^ (SD = 4.5), with 46.6% being graduate students.

### 2.4. Study 2: Jogging

In Study 2, the 88 participants had a mean age of 21.9 (SD = 2.4) years. As shown in Table 1, most participants identified as Non-Hispanic White (68.2%), while others were Non-Hispanic Black (25.0%), other Hispanic (1.1%), and other/multiracial (5.7%). With regards to gender, the sample was almost evenly distributed (45.5% males and 54.5% as females). The mean measured body mass index (BMI) of the sample was 24.2 kg/m^2^ (SD = 4.2). Again, participants were randomly assigned to one of four different conditions of 22 participants each: (a) jogging before memory encoding, (b) no exercise, (c) jogging during memory encoding, or (d) jogging after memory encoding.

### 2.5. Materials

Participants in both Study 1 and 2, completed a demographic survey at baseline (prior to the exercise stimulus).

#### 2.5.1. Retrospective Memory

Also for participants in both studies, memory (retrospective memory) was assessed with the standardized Rey Auditory Verbal Learning Test (RAVLT) [32], using procedures put forth in the test manual. Participants were asked to listen to and immediately recall a recorded list of 15 words (List A) five times consecutively (Trials 1–5). They were then asked to listen to and immediately recall a list of 15 new words (List B). Performance recalling list B was the primary outcome of interest in this study, with List A serving to induce proactive memory interference. Immediately following the List A recall trials, participants were required to recall the words from List B (Trial 6).

#### 2.5.2. Exercise

We monitored heart rate (Polar; FT1) continuously during exercise, with participants in both Study 1 and 2 asked to select an appropriate pace after researchers provided the following instructions:

“*Please select a pace similar to one you would choose if you were late to class. Thus, it will not be a leisurely walk. Nor will it be a run.*”

Participants in Study 1 walked on a Woodway treadmill for 15 min and were then asked to indicate their Rating of Perceived Exertion (RPE) using Borg’s (6–20) RPE scale [33] during their exercise. Participants in Study 2 were asked to jog on a treadmill for 15 min, with the first 5 min at an easy self-selected jogging intensity (keeping the pace at an RPE of 11–12), the next 5 min at a self-selected faster pace (keeping the pace at an RPE of 13–15), and the last 5 min at a self-selected hard pace (keeping the pace at an RPE of 16–20). This progressive exercise intensity was employed to ensure that all participants reached the same relative high-intensity by the end of the exercise bout. Thus, Study 2 participants jogged on a Woodway treadmill for 15 min and were also asked for their RPE using Borg’s 6–20 RPE scale [33] during the mid of the 1st 5 min set, mid of the 2nd 5 min set, and immediately after the 15 min.

### 2.6. Statistical Analysis

Data were analyzed using Stata v14 (StataCorp LLC., College Station, TX, USA). We conducted chi-square tests on the categorical variables (i.e., sex, race, education) to assess any group differences in these participant characteristics. In each study, we used one-way analysis of variance (ANOVA) to compare all four groups on all continuous variables (i.e., age, BMI, and % of maximum HR) and to determine if there were any group differences on these variables for performance on List B (Trial 6).

## 3. Results

**Figure 1:**
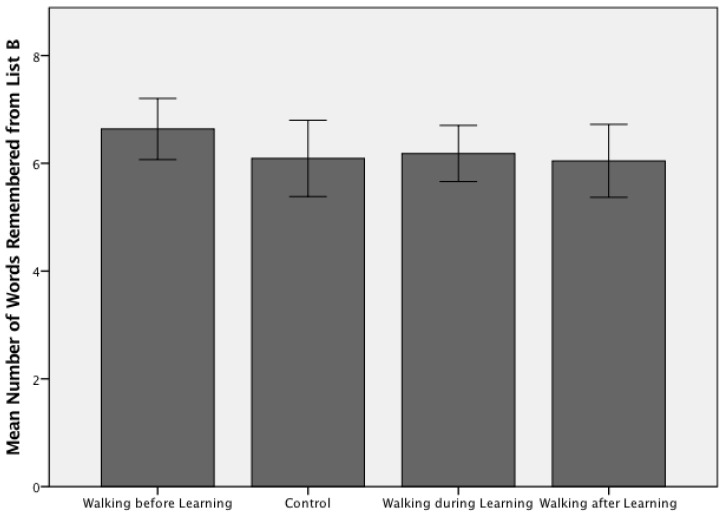
Free recall scores for Trial 6 (List B) across the four experimental arms for Study 1 (*n* = 88; walking and learning). A one-way ANOVA confirmed there were no significant group differences between the temporally varied exercised and no-exercise control conditions (F(3, 84) = 0.758, *p* = 0.521), with an average recall score of 6.24 (SD = 1.45) words (range 3–10).

**Figure 2:**
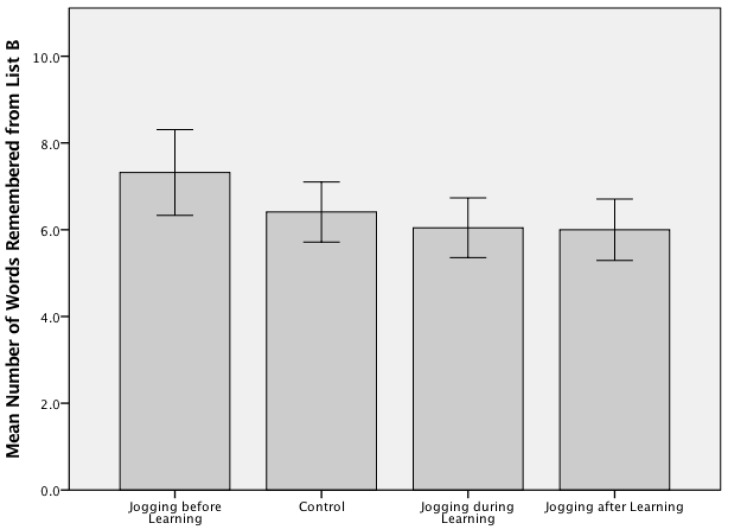
Free recall scores for Trial 6 (List B) across the four experimental arms for Study 1 (*n* = 88; jogging and learning). A one-way ANOVA confirmed there were no significant group differences between the temporally varied exercised and no-exercise control conditions (F(3, 84)= 2.5, *p* = 0.068) with an average recall score of 6.44 (SD = 1.88) words (range 3–12).

The average number of words recalled correctly by participants in the exercise before encoding group was higher than all other groups for both Study 1—Walking and Learning (6.64 ± 1.33 words; Figure 1) and Study 2—Jogging and Learning (7.318 ± 2.32 words; Figure 2). A one-way ANOVA confirmed there were no significant group differences between the temporally varied exercised and no-exercise control conditions (F(3, 84) = 0.758, *p* = 0.521) in Study 1. Participants in Study 1 remembered an average of 6.24 (SD = 1.45) words across all four groups, with a range of 3–10 correctly recalled items. Participants in Study 2 remembered an average of 6.44 (SD = 1.88) words across the four experimental groups, with a range of 3–12 correctly recalled items. A one-way ANOVA demonstrated there were no significant group differences between the included experimental conditions (F(3, 84) = 2.5, *p* = 0.068) in Study 2.

## 4. Discussion

No study, to our knowledge, has previously examined the effects of exercise on reducing PI. Our main finding was that participants who exercised before memory encoding, compared to other temporal periods and no exercise, had the highest absolute number of words recalled, though this was not a statistically significant finding, and a tendency toward this finding was only evident among those participants engaged in moderate to high intensity exercise (i.e., jogging versus walking).

Neuroimaging studies corroborate the crucial role of the PFC in verbally processing novel information, which is deemed to be especially vulnerable to PI distortion [5,6,7,8,9,10]. In theory, exercise might help to reduce PI, perhaps by inducing PFC activation and allocation of cognitive resources [22,30]. We observed no convincing evidence that exercise can reduce PI, and as a result, help enhance memory recall. However, our second experiment (jogging) produced suggestive evidence that jogging before memory encoding might prove beneficial (small effect size) in a study with a larger participant sample size, indicating that future work in this line of inquiry is warranted.

It has been reported that the ability to form distinctions is particularly important for recall accuracy [34]. Thus, future work might systematically alter stimuli distinctiveness to evaluate whether different levels of distinctiveness facilitate learning capacity relative to PI. In addition, the primacy and recency components of word lists are known to be robust to interference, availability, and processing issues that could undermine later recall [35], and future work should consider evaluating this factor for added clarity; especially, as indicated previously, the shorter the retention interval, the less likely PI will play an appreciable role in short-term memory processing. We also suggest that future studies on this topic employ the AB/AC paradigm, which is the scientific gold standard lexicon for assessing PI [36].

It is important to continue emphasizing this novel line of research. Science is just beginning to assess the specific influence of exercise timing and intensity on memory encoding and consolidation. Past work suggests exercise-associated arousal may exert an advantageous effect on both encoding and consolidation [20]. Although, it has also been argued that tasks requiring delayed, but not immediate free recall, are more susceptible to the beneficial influences of physical and cognitive arousal [37]. A potential consideration for subsequent research is to recruit a larger sample size to ensure that each group is well-representative of the broader college-student population, or, perhaps, to replicate this experiment employing a within-subject design. As limited research has been published on this topic, this experiment presents an exciting opportunity for continued scientific discourse and investigation regarding exercise and PI.

## Figures and Tables

**Table 1:** Demographic characteristics between study 1 and study 2.

	Study 1 (*n* = 88) Walking	Study 2 (*n* = 88) Jogging
Age (years)	M = 22.3 ± 3.7	M = 21.9 ± 2.4
Body Mass Index (kg/m^2^)	M = 25.3 ± 4.5	M = 24.2 ± 4.2
Sex		
Male	52.3%	45.5%
Female	47.7%	54.5%
Race		
Caucasian/Nonhispanic White	65.9%	68.2%
African American/Nonhispanic Black	20.5%	25.0%
Other Hispanic	-	1.1%
Other/multirace (Asian)	13.6%	5.7%
Speed (mph; *n* = 66)		
Walking	M = 3.2 ± 5	-
0–5 min into jogging	-	M = 4.6 ± 9
6–10 min into jogging	-	M = 5.7 ± 9
11–15 min into jogging	-	M = 7.0 ± 1.3
Heart rate		
Resting (*n* = 88)	M = 73.5 ± 13.1	M = 71.9 ± 10.9
7.5 min into walking/jogging (*n* = 66; control group omitted)	M = 113.3 ± 21.1	M = 163.6 ± 16.5
14 min into walking/jogging	M = 116.0 ± 20.1	M = 183.7 ± 12.9
5 min after walking/jogging	M = 80.6 ± 13.7	M = 106.4 ± 15.8
Rating of perceived exertion		
7.5 min into walking (*n* = 66; control group omitted)	M = 9.1 ± 1.4	-
14 min into walking	M = 11.9 ± 14.5	-
0–5 min into jogging	-	M = 11.4 ± 7
6–10 min into jogging	-	M = 14.7 ± 1.2
11–15 min into jogging	-	M = 18.7 ± 9
